# A Commentary on Blue Zones^®^: A Critical Review of Age-Friendly Environments in the 21st Century and Beyond

**DOI:** 10.3390/ijerph18020837

**Published:** 2021-01-19

**Authors:** Hannah R. Marston, Kelly Niles-Yokum, Paula Alexandra Silva

**Affiliations:** 1 Health & Wellbeing Strategic Research Area, School of Health, Wellbeing & Social Care, The Open University, Milton Keynes, Buckinghamshire MK7 6HH, UK; 2 Department of Health and Public Management, College of Business & Public Management, University of La Verne, La Verne, CA 91750, USA; kniles-yokum@laverne.edu; 3 Centre for Informatics and Systems (CISUC), Department of Informatics Engineering (DEI), University of Coimbra, 3030-290 Coimbra, Portugal; paulasilva@dei.uc.pt

**Keywords:** ageing, age in place, community, Coronavirus, COVID-19, gerontechnology, human centred design, older adults, rural planning, technology, smart ecosystem, smart islands

## Abstract

This paper explores the intersection of the World Health Organization’s (WHO) concepts of age-friendly communities and The Blue Zones^®^ checklists and how the potential of integrating the two frameworks for the development of a contemporary framework can address the current gaps in the literature as well as consider the inclusion of technology and environmental press. The commentary presented here sets out initial thoughts and explorations that have the potential to impact societies on a global scale and provides recommendations for a roadmap to consider new ways to think about the impact of health and wellbeing of older adults and their families. Additionally, this paper highlights both the strengths and the weaknesses of the aforementioned checklists and frameworks by examining the literature including the WHO age-friendly framework, the smart age-friendly ecosystem (SAfE) framework and the Blue Zones^®^ checklists. We argue that gaps exist in the current literature and take a critical approach as a way to be inclusive of technology and the environments in which older adults live. This commentary contributes to the fields of gerontology, gerontechnology, anthropology, and geography, because we are proposing a roadmap which sets out the need for future work which requires multi- and interdisciplinary research to be conducted for the respective checklists to evolve.

## 1. Introduction

There is a growing body of scholarly research [1,2] exploring how urban ageing impacts towns and communities in the Western world while taking an age-friendly approach based on the World Health Organization’s (WHO) framework published in 2007 [3]. For nearly twenty years, scholarly research [1,2,4,5,6,7,8,9,10,11] has illustrated how many towns and communities have been working towards ensuring their respective environments include age-friendly features.

At the time of publication (2007), the age-friendly framework proposed by the WHO [3] provided a checklist that offered and afforded academics and policy-makers the opportunity to adapt key facets within their own environments. However, scholarly interest started to grow in the 1990s and at the beginning of the 21st century with regard to technology use by older people [12,13,14,15,16,17,18,19,20,21,22,23,24,25,26,27,28].

Moreover, research from the standpoint of gerontology and age-friendly cities and communities is limited to the domain of Blue Zones^®^. To date, existing research is taken from the standpoint of epidemiology ranging from dietary [29,30,31,32,33,34,35], depression and mental health [36], health, cardiovascular and heart disease [37,38,39,40,41,42], longevity [43,44,45,46], obesity, and physical activity [47] perspectives. Furthermore, while academe has known about Blue Zones^®^ for approximately 20 years, acknowledging that there is something special about these areas, there is a paucity of scholarly research from the social sciences stance. However, different Blue Zones^®^ characteristics have not been incorporated into the WHO framework published in 2007 [3].

In this commentary, we aim to discuss Blue Zones^®^ and how features of the age-friendly framework posited by the WHO [3] and the smart age-friendly ecosystem (SAfE) framework posited by Marston and van Hoof [48] can be considered for future integration into such environment(s). In particular, we aim to explore and understand how the respective frameworks [3,48] could be integrated in a variety of ways and settings to produce recommendations and notions for future work that could lead to the development of a contemporary framework specifically aimed at incorporating both the Blue Zones^®^ concept and age friendly community principles, offering appropriate interventions and applicable solutions.

The work presented here is significant because it contributes to the fields of gerontology, geography, social sciences, social policy, industry, technology, and health. Furthermore, the work presented in this paper has the potential to impact societies on both national and international scales as it discusses the WHO age-friendly framework, which for 12 years has been used primarily as a marker for towns and cities in the Western world. Moreover, the smart age-friendly ecosystem (SAfE) [48] framework was posited by taking a case study approach using Milton Keynes, United Kingdom, as an exemplar, given the existing notions of a “new town”, “smart city”. This commentary aims to offer readers a framework as a means of forming an initial basis for future research, case studies and explorations, with a view to enhancing, developing, and improving the blueprint over time.

The content of this commentary is novel in that it bridges the gaps in existing literature from the field of gerontology whereby to date there is a paucity of literature surrounding Blue Zones^®^ and their relationship(s) with the age-friendly cities and communities domain, digital technologies including the relationship(s) and connectors of digital technologies and Blue Zones^®^.

The outline of this commentary presents an overview of Blue Zones^®^, and contemporary literature surrounding Western Blue Zones^®^ sites. Furthermore, in Section 3, we present the respective Blue Zones^®^ checklists (Home, Kitchen, Bedroom, and Tribe) and in Section 4 we critically review/analysis of the four checklists. Section 5 discusses the features surrounding the WHO age-friendly and the smart age-friendly ecosystem (SAfE) frameworks. The discussion and conclusion—Section 6—sets out a roadmap for moving this debate forward and proposes recommendations for future steps.

## 2. Blue Zones^®^

In this section, we will explore what is meant by Blue Zones^®^ and existing relevant research.

### 2.1. What Is a Blue Zones^®^?

The history of the Blue Zones^®^ stems from the founder Dan Buettner, a National Geographic Fellow [49], who has to date discovered five places across the world labelled as Blue Zones^®^: 1. Okinawa (Japan), 2. Ogliastra Region, Sardinia (Italy), 3. Nicoya Peninsula (Costa Rica), 4. Ikaria (Greece), and Loma Linda (California).

Blue Zones^®^ are places or regions which have a high concentration of centenarians in addition to clusters of people who have reached old age without disease and/or other health conditions such as obesity, cancer, diabetes, and heart problems [49]. Furthermore, Buettner [49] notes how individuals living in these areas or regions not only live longer, but their day-to-day lives are fulfilled with activity, citizens who experience good health, and positive engagements with their families and communities. A Blue Zones^®^ team includes a myriad of team members who are anthropologists, dietitians, demographers, epidemiologists, and medical researchers. However, experts from the field of gerontology do not seem to be included [50].

Buettner and his team identified nine commonalities in four categories and practices across the five Blue Zones^®^ regions and features in the Blue Zones^®^ Solution [49]. These are: move—1. move naturally; right outlook—2. purpose and 3. downshift; eat wisely—4. 80% rule, 5. plant slant, and 6. wine at five; connect—7. right tribe, 8. loved ones first, and 9. belong. These factors are characterized as the Power of 9 and form a triangle, with move naturally at the top, and the notion of belong and the three factors, commonalities or practices forming the base [47,49].

A contemporary piece of research conducted by Riddell [50] puts forth the perspective of urban planning from the standpoint of the USA exploring and identifying correlations between urban planning and design, the physical environment, health, and wellbeing.

Furthermore, there have been additional experiments and explorations across the USA in an attempt to redevelop the areas/regions of Minnesota, California, and Iowa, implementing a Blue Zones^®^ ethos. However, with the exception of Loma Linda—located in California—the other four Blue Zones^®^ were created organically, isolated from their respective mainland regions. Riddell [50] highlights the completed projects across the USA which include: 1. Albert Lea—Minnesota, 2. California Beach Cities—a. Manhattan Beach, b. Hermosa Beach, and c. Redondo Beach, and finally 3. State of Iowa. Furthermore, there are four additional areas which are planned for re-engineering these regions into Blue Zones^®^ and include, 1. Honolulu and Hilo—Hawaii, 2. Wisconsin, 3. Indiana, and 4. Klamath Falls—Oregon [50]. Below, we describe two of the experimental projects to understand how the Blue Zone concept, which was organically created in isolated regions, has been transferred to different regions of the USA.

### 2.2. Albert Lea—Minnesota

Albert Lea was the first region for Buettner [49] sought out for the Blue Zones^®^ experiment, consulting public health officials at the University of Minnesota who in turn requested Buettner to measure and assess each campaign [50]. This town, as Riddell [50] notes, was chosen because it represents a “typical” American city, comprised of ~17.5 K residents [51], which was not too large nor was it too small and therefore could be a model for other regions and cities across the state and the country [50]. Moving forward, the Blue Zones^®^ team chose a 20-mile “life radius” around homes and workplaces because this is where the main activity is conducted during the day [50].

Substantial financial support was provided by the American Association of Retired Persons (AARP), and the University of Minnesota—School of Public Health also joined the pilot project to assist the Blue Zones^®^ team with their assessment of Albert Lea in 2009 [50]. A series of walking groups were formed and met up several times each week to answer whether the environment promoted walking [50]. Such groups, as Riddell [50] notes, are similar to the support groups or *Okinawa’s moai* concept which affords physical activity by moving naturally and interacting with the right tribe, coupled with the sense of community. Another creation was developed and included the “walking school bus” which facilitated both parents and children who would usually use the bus to actually walk together to school. Riddell [50] highlights this notion and activity because of its popularity, resulting in older adults volunteering to walk with the “walking school bus”. Considering the Power of 9, this approach and implementation engaged several factors, resulting in greater physical activity by all residents, community spirit, and a sense of purpose for the older adults when helping within their own community [49,50].

Both the natural and built environments were analysed, which included grocery stores, schools, restaurants, and included questions relating to the type of food available/displayed, whether the environment was walkable, and whether there were attractive public green spaces being used. Similar approaches have been conducted by van Hoof and colleagues [2] who conducted an assessment based on the age-friendly features in the Dutch municipalities of The Hague and Zoetermeer. By employing a qualitative photoproduction approach based on the Checklist of Essential Features of Age-Friendly cities [3], five neighbourhoods were assessed. Across the two municipalities, large visual representations were identified within five of the eight domains of the WHO age-friendly mode: 1. communication and information; 2. housing; 3. transportation; 4. community support and health services; 5. outdoor spaces and buildings [2,3]. The next area to be explored and assessed by the Blue Zones^®^ team was the built environment which included areas such as grocery stores and their respective layouts. Additionally, the built environment also encompassed policy and budgetary areas which impact on the overall region or city. The Blue Zones^®^ team identified in the grocery stores how healthy food products were not at eye level and instead were placed away from direct eye contact. Yet, unhealthy food products were clearly visible, placed at the checkout areas and on tables [49,50]. Moreover, the Blue Zones^®^ team rearranged the produce throughout the stores, replacing sweets that were visible at the checkout points with fruit and nuts, followed by highlighting sweet potatoes and beans to the consumer with specific “Blue Zones labels” to signify the healthiness of the produce to the consumer [49,50]. Within the school environment, changes to snacks were also introduced and replaced with healthy options in vending machines, replacing crisps, biscuits, and fizzy drinks [49,50]. Buettner [49] highlights that the final stage within this town was working with the residents themselves and at a meeting comprised of 4000 people who pledged to become involved with the project and commenced restocking their own larders and house appliances with healthier food [49,50].

Riddell [50] purports that these changes at various levels of the community from the built environment to the individual ecosystems led to an overall positive change, by employing a three-pronged approach of community, public and private engagement, and partnership. This also included key leadership within the city such as the mayor, presidents of commerce, and educational superintendents, coupled with support and interest from the media, investing various energies into the project for the overall benefit of the town [49,50].

The impact of these changes across various intersections of the community from the home ecosystem, educational environments, and the wider built environments, including community support groups such as the walking groups, as Riddell [50] notes, led to substantial positive health benefits. The impact witnessed a 40% reduction associated with healthcare costs and 12,000 pounds lost (in weight) [49]. Further community and organizational changes were employed in the workplace, although Riddell does not state exactly what changes were made to these restaurants and workplaces.

### 2.3. State of Iowa

This initiative was rolled out in 2011 to support the State of Iowa to become a healthier state and resulted in the it becoming a demonstrator site. This included twenty towns used to create healthy living environments. One town—Spencer—was comprised of around 11,233 residents [52]. As Riddell [50] notes, this town found the challenge difficult (p. 58) because there was limited leadership and employment positions which resulted in assistance from Alberta Lea and the California beach cities. Riddell [50] notes how the size of this town, coupled with the importance of community engagement, motivation, and spirit, were crucial building blocks to bridge closer relationships [50].

However, between commencing this challenge, over a two-year period, Spencer town was named and granted the first certification for the Blue Zones^®^ community [50] (p. 59) and, as noted in the previous section, included the integration and formed part of the Power of 9 concept [53,54], and fresh fruit and vegetables formed the ethos and activity of the Blue Zones^®^ region. In Spencer town, a total of 36 community plots were created, enabling residents in the community to access fresh produce. These community plots facilitated additional factors within the Power of 9, including moving naturally, plant slant, the right tribe, and a sense of connection and belonging to the community and loved ones first. Riddell [50] also notes how this community created walking moais which facilitated and integrated additional factors surrounding the Blue Zones^®^ ethos of healthy behaviour and happiness within the social and urban environments [53,54]. In the following section, the four Blue Zones^®^ checklists are presented.

### 2.4. Blue Zones^®^ Checklists

A Blue Zones^®^ checklist is available through membership which offers individuals a tool to understand their home environment, social network, and guidance for improvement [55]. Below is an overview of each of the items of the checklist: 1. Home, 2. Kitchen, 3. Bedroom and 4. Tribe.

### 2.5. Home Checklist

The *Home* checklist [56] relates to various aspects and activities within the home environment and includes access to weighing scales to enable a person to weigh themselves daily, owning one television, the removal of power tools and instead using hand operated appliances, having the space to grow vegetables, owning a dog for companionship, and conducting physical activity through various and different forms of exercise. Additionally, owning additional transportation such as a bicycle has the potential to encourage regular exercise, as well as owning a variety of sports footwear and equipment (e.g., basketball, baseball, football, golf balls and clubs, inline skates, camping supplies, and running shoes) to motivate additional physical activity. Additionally, it is suggested that growing indoor plants will assist with exercise while maintaining the health of the plants; further space is needed to create a “destination room” which is a popular room in the home, and affords supplementary exercise by climbing the stairs, as well as disconnecting the automatic garage door to encourage a person to get out of their car and open the door. Removing the television remote would enable additional movement when changing the channel, while placing cushions on the floor to facilitate strength training of the thighs, glutes, and lower back.

This 13-item checklist in Table 1 displays a range of questions, answers and points relating to the respective answer(s), enabling a person to gain a maximum of 55 points. Table 1 displays the item checklists, the answer(s), points, and the purpose/additional information that a person can read and learn from.

Upon completion of the checklist the person can calculate their final score and review it to understand where they need to improve (Table 2).

#### 2.5.1. Kitchen

The Kitchen checklist [57] suggests a person should place snacks into small bags, move the fruit and vegetables to the front of the fridge, while reducing the size of crockery and glassware in a bid to reduce consumption/overeating. Kitchen cupboards (e.g., a specific drawer for junk food) should be organised and all digital devices (e.g., television) should be removed from this environment. While the checklist suggests fruit should be placed at eye level to encourage healthier eating habits, mechanical kitchen appliances should be removed and replaced with hand operated ones. Table 3 displays the 10-item checklists, a range of answers and points relating to the respective answer(s), and the purpose/additional information that a person can read and learn from.

Upon completion, the person can calculate their final score and review it to understand where they need to improve (Table 4), enabling a person to gain a maximum of 40 points.

#### 2.5.2. Bedroom

The Bedroom checklist [58] relates to one’s sleep patterns, and the comfort of the person’s bed/mattress and/or pillows, while it is suggested the room temperature should be set to a specific temperature, and the ambience (e.g., lights) should be considered. Individuals should consider removing digital devices (e.g., television, computers, alarm clocks) from this space and to facilitate relaxation a person should consider introducing lavender. Additionally, windows should be larger. This 11-item checklist, presented in Table 5, displays a range of answers and points relating to the respective answer(s), enabling a person to gain a maximum of 45 points, in addition to supplementary information relating to each item.

Once a person has completed their checklist for the bedroom environment, they can calculate the number of points they have gained and review Table 6 to understand how this specific environment is enriching their health, wellbeing, and lifestyle.

#### 2.5.3. Tribe

The Tribe checklist [59] relates to a person’s lifestyle activities and behaviours, their beliefs, their social networks, their weight, their self-perceived happiness, and feelings of loneliness and social isolation. This 10-item checklist, displayed in Table 7, displays a range of answers and points relating to the respective answer(s) enabling a person to gain a maximum of 65 points. Unlike Table 1, Table 3, and Table 5, there is no explanation to a person about their total score—instead, the person is required to submit their scores online and a person completing this also has the option to include friends who can also answer the same questions.

It is worth noting that, on the website, there is an option for additional calculations for 1–2 friends, using the same questions above. For the *Tribe* checklist, the person can gain a maximum of 65 points. There is no additional information relating to the total score. However, the person can submit their scores and also print out the related information, enabling the person to look at areas of where they need to improve. In the following section, we provide a critical review of the four checklists presented above.

## 3. Critical Review

In this section, we provide a critical review/analysis of the four checklists presented in the previous section. To conduct this review/analysis we draw on the work by Munthe-Kaas and colleagues [60] who conducted a systematic mapping of 25 checklists in a bid to assess transferability.

In the respective review, Munthe-Kaas et al. [60] propose nine themes as a way of evaluating content analysis of checklists. In the following sections, we present each of the four Blue Zones^®^ checklists (Home, Kitchen, Bedroom and Tribe) and their viability of transferability against the respective themes: 1. Population, 2. Intervention:, 2a, Intervention characteristics, 2b: Intervention delivery, 3: Implementation Context: 3a. Service providers (individuals), 3b. Implementing organization, 4. Comparison intervention, 5. Outcomes, 6. Environmental context, and 7. Researcher conduct, proposed by Munthe-Kaas et al. [60].

### 3.1. Overview of Commonalities across Checklists and Analysis

Reviewing all of the checklists against the first theme, *Population*, there is no specific information and/or context presented associated with the respective populations and their characteristics. Munthe-Kaas et al. [60] note that this theme does not only include demographic information but also additional attributes such as health conditions, illness, the acceptability, or reception of the respective checklists by users/subscribers of the checklists, their respective location (e.g., country, state/county, physical space), personnel support, and/or social networks.

### 3.2. “Home”—Critical Review

In Table 8, the transferability of the “Home” checklist across the 9 themes is limited. Primarily, the items in this checklist are associated with physical activity, weight loss, and tranquillity. However, this checklist and its respective descriptions (Table 1) do not contextualise various populations environments. For example, Item 4—“Grow and maintain your own garden”—aims to facilitate healthy and light physical activities and living. Yet, it assumes that everyone has a garden or at least access to a garden to potentially grow vegetables. This is not the case for many people across the life course, in particular those who live in inner-city housing, who may not even have a balcony, let alone green space (e.g., allotment) to grow their own vegetables.

Item 9, “Create a destination room”, assumes that the person will live in a home that affords the luxury of creating a tranquil space. However, for many people, they do not have the space to create a “destination room” and some people choose (or have no other option due to their financial status) to live in a single-story environment (e.g., apartment). Additionally, this checklist does not acknowledge multigenerational living or adults who are ageing without children (AWOC) [61,62,63,64]. These two forms of living also impact on the home space and can change quickly—be it through ill health or chaos. Furthermore, this type of societal living arrangement impacts the home environment considerably and, as we move forward into the 21st century, this should be reflected in future iterations of frameworks and domains (e.g., gerontology, gerontechnology, planning, urban design, and social sciences).

Finally, the “Home” checklist makes some assumptions based on individual and environmental circumstances (Table 8). For example, it assumes that an individual lives in an environment that allows pets, has space for a garden, has a garage, and a separate space for an exercise area. From a physical perspective, not everyone can sit on the floor, or can use hand tools, or practice the sports listed here. As previously noted, information surrounding population characteristics is sparse and does not reflect the respective circumstances of a person’s living situation.

### 3.3. “Kitchen”—Critical Review

In Table 9, the transferability of the “Kitchen” checklist across the 9 themes is limited. Primarily, the items in this checklist are associated with white goods (e.g., fridge), weight loss, and physical space in the living environment. However, this checklist and its respective descriptions (Table 3) do not contextualise various populations environments.

For example, Item 3—“Only own dinner plates that are 10” or smaller”—aims to reduce portion sizes and is set within an American context. There is no information relating to other eating and lifestyle habits surrounding populations living in different continents (e.g., Europe, Asia, etc.). Item 5, “Create a junk food drawer”, suggests hiding junk food implying it is more likely to be consumed when visible. However, there is a lack of acknowledgement of prospective multigenerational living circumstances and an assumption that the primary aim is healthier eating/weight loss. Additionally, Item 6—“Pre-plate your food”—aims to reduce and avoid “eating family style by leaving the serving dishes on the counter”. As noted in the description (Table 3), if a person is hungry and wants additional servings they can walk to the counter and reduce the temptation to automatically have a second serving. Item 9, “Use hand operated kitchen appliances”, assumes that people have the dexterity in their hands to manually use kitchen appliances. However, for some people with chronic illnesses, health conditions and disabilities, using manual appliances is not possible; this is not reflected nor considered in the additional information provided against this item. As previously noted there is no information relating to or associated with population characteristics, suggesting that items such as Item 9 have the potential to alienate many people across the life course from engaging with such a checklist. Item 10, “Place a longevity food list on your refrigerator”, has the potential to be adapted for people who may have smart fridges and/or who shop online. For example, while such a list can enable people to remember the good and bad foods to have in their diet, when stocks are getting low, there is the potential to enable Internet of Things (IoT) appliances [65,66] to reorder. Similarly, if a person uses online shopping as their primary method of shopping, then they are able to add such items to their shopping list in preparation for their next delivery.

The “Kitchen” Checklist assumes that an individual lives in an environment whereby they have a kitchen, the person has the space to allow for extra items such as bowls for fruit which in turn can be placed on the countertop. From a physical perspective, not everyone can operate hand appliances or owns appliances, or can reach the top shelf of their refrigerator—if they own one. There are many instances where individuals do not have much choice about the food they have on hand or consume.

### 3.4. Bedroom Critical Review

As displayed in Table 10, the transferability of the “Bedroom” checklist across the 9 themes is limited. Primarily, the 11 items in this checklist are associated with technology (e.g., TV/computer), physical space and household items (e.g., curtains), temperature, ambience, and bedding.

Item 1, “Know your snore score”, relates to one’s sleep patterns and habits. In this item, additional information is provided by various sleep associations within the context of America. However, and unfortunately, there is no scholarly work cited to support this item. Furthermore, there are additional questions for people to consider and to follow up, on their own accord (Table 5). Item 2, “Own a comfortable mattress and comfortable pillows” aims to facilitate positive sleep which in turn has the potential to translate into greater productivity and improve overall health and wellbeing. However, purchasing a good mattress is not cheap and this may be beyond many people’s financial means. Additionally, while a mattress may or should be replaced nearly every decade, this too can become expensive and wasteful from the standpoint of recycling and sustainability. Item 3, “Set the temperature in your bedroom to 65 °F at night”, does not account for various temperature differences found in different continents (e.g., Scandinavia) or the type of materials and age of the housing when built. For some people who live in housing that has been poorly built and insulated, heating would be required during the colder months and may not have the option of setting a specific temperature. For some people who live in housing that is historical, placed in a conservation area and is generally 100–200 years old, heating a room to a certain temperature is not possible because they would need to ensure the temperature is appropriate for them to go to bed, reducing damp and potential health issues resulting from a cold room/living space. However, with recent innovative technologies such as the IoT, there is the option to set individual rooms within the living space to different temperatures that can be set prior to one coming home from work or other outdoor activities [67]. Similarly, this type of technology can also be used for lighting (Item 4) on the Bedroom checklist, and via the various products available on the market, dimming lights can be set for various times of the evening and of the day. For more information relating to IoT devices implemented into real world settings, see Marston and van Hoof [48], and Marston et al. [68,69,70,71]. Item 10, “Install double paned windows in the bedroom”, assumes users of this checklist own their own home. For people who are renting, which can be more common in European countries and outside of the USA, installing a double paned window may not be possible because of the tenancy agreement and other respective regulations.

Finally, the “Bedroom” checklist assumes that an individual has access to a separate bedroom coupled with the choice of where and how they sleep or rest. Some bedrooms do not have windows and even if they do, not every person can afford to put in new windows. Additionally, a remote control may sometimes be the only option a person has to operate their television and, given the phenomenal rise of smart TVs, the notion of getting up and walking to the television to change a channel may simply not be possible. Furthermore, lighting, temperature, and alarm clocks may not be a matter of personal preference but rather a matter of personal safety, and as noted above, the IoT can afford individuals the opportunity to take control of lighting, temperature, and safety on their own.

### 3.5. Tribe Critical Review

Table 11 highlights the questions posed in the “Tribe” checklist [59] (Table 7) and analysis of the checklist based on the Munthe-Kaas et al. [60] framework highlights two primary themes: *outcomes* and *environment*. The wording of the items from this checklist is different compared to the other checklists, requiring the individual to self-report or to report on someone else’s behalf. Depending on who is completing this checklist, a complete overview may not be ascertained because if a third party is completing the checklist on behalf of someone else, they may not know how many units of alcohol that person drinks, or whether they take part in religious activities, or their level of happiness, or whether they feel lonely, etc.

The “Tribe” checklist [59] assumes that an individual lives in an environment that allows for physical exercise and can participate in something fitting the definition of exercise; other items included religious activities and social participation, including the number of individuals in one “Tribe” should be viewed from a variety of situational circumstances including preferences. There is a clear distinction to being “lonely” and being “alone”.

### 3.6. Summary

There is little theoretical underpinning associated with the items presented in each of the four checklists in addition to a paucity of research supporting the evaluation of the checklists. As noted in the previous sections, the four checklists seem to be posed in the context of the USA with little consideration for other citizens located in different continents. Furthermore, additional consideration and questioning should be considered when aligning these checklists with the five Blue Zones^®^ and whether there would be similar mapping outcomes. Given the healthy ageing narrative, the concept of incorporating a life course perspective for residents/citizens within the Blue Zones^®^ and who live in other countries and who may be accessing the checklists for their own personal use is needed to fully gauge the understanding and respective situation of a person. Additionally, with this paucity of theoretical underpinning coupled with the notion of implementing a life course perspective, there is the possibility of facilitating actors to capture and complement future data collections, including qualitative data including observations, diaries, and narratives from all citizens not just older people. Access to healthcare, socioeconomic status, age, physical ability, and other factors play important roles in fully understanding one’s personal and environmental circumstances.

Our critique of these checklists is intended to widen the perspective of diversity and of the human experience related to older adults and persons with different levels of ability, such as those with chronic health conditions, disabilities, or dementia. Additionally, consideration of the variety of environments in which individuals live is critical for an inclusive approach. In the following section, we explore age-friendly frameworks and approaches that may assist future iterations of Blue Zones^®^ checklists [56,57,58,59] and bridge future developments in these two domains.

## 4. Theoretical Approaches and Frameworks to Age-Friendly Cities and Ecosystems

To date there has been a wealth of research surrounding age-friendly cities and further reading can be found via the works of Marston and van Hoof [48], Marston and Samuels [67], Marston et al. [68], Buffel et al. [7,10,72], and van Hoof et al. [2,6,73], which provide an extensive overview of literature surrounding age-friendly research.

**Figure 1:**
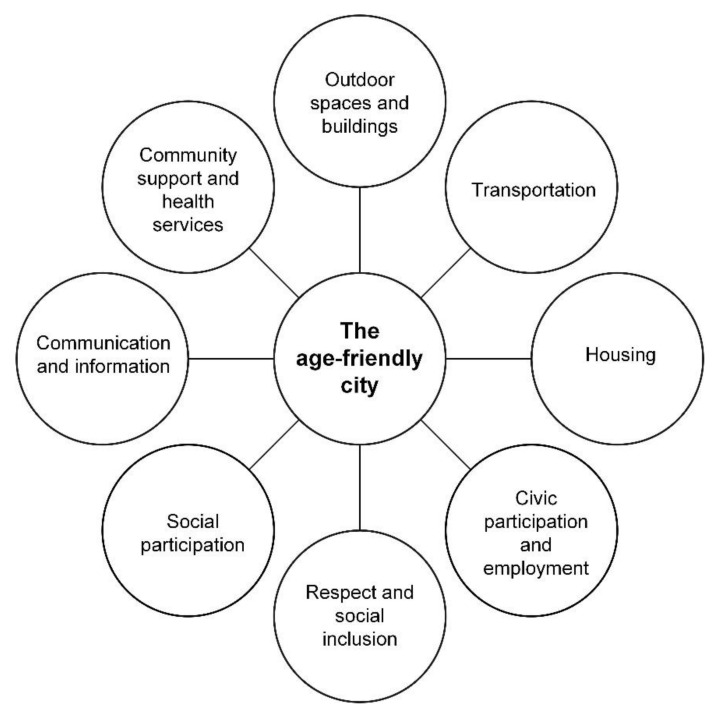
The eight domains of an age-friendly city [3].

**Figure 2:**
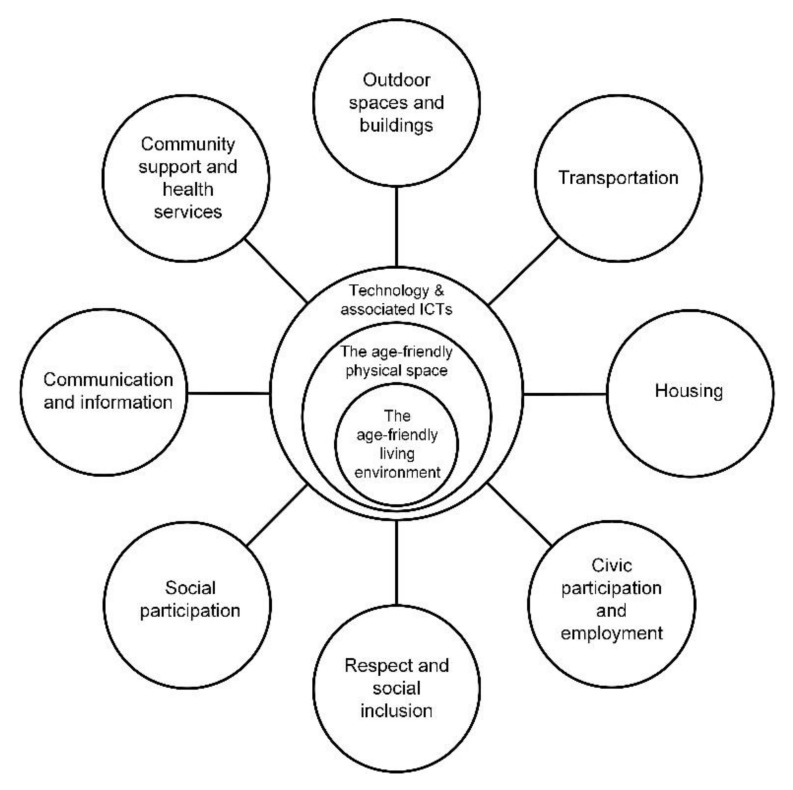
The Smart Age-friendly Ecosystem (SAfE) framework [48]. Permission granted by Drs Marston and van Hoof.

However, the existing work surrounding this framework has been conducted by Buffel et al. [72], Plouffe and Kalache [4], and Rowles [74] who have previously described the historical efforts of the WHO in a bid to positively participate, connect, and support different cities and communities in the remit of the WHO age-friendly initiative in converting respective cities and communities through development processes and following the “Checklist of Essential Features of Age-Friendly Cities” [3] to become more “age-friendly”. With this growing body of scholarly work which discusses the WHO age-friendly framework [3] (Figure 1) in conjunction with a recent extended version to this framework proposed by Marston and van Hoof [48] (Figure 2), this contemporary research has identified novel areas for bridging gaps in the literature and working in multi- and cross-disciplinary teams to advance the narrative of this domain.

Figure 1 illustrates the eight domains of the framework, making up the original notion of an “age-friendly” environment/community.

However, as noted extensively by Marston and van Hoof in 2019 [48], 13 years after the original framework was published, there has not been any additional iterations coinciding with societal changes such as technology use, deployment, and implementation. This is where the proposed extension—the Smart Age-friendly Ecosystem (SAfE) framework—was created and introduced [48]. Figure 2 illustrates the new framework acknowledging the physical space, technology, and associated ICTs (information communication technology) as described by Marston and van Hoof [48].

The SAfE framework illustrates the relationship technology has with various domains across different segments and interconnections within our respective cities and communities. The physical space, as posited by Marston and van Hoof [48], refers to and acknowledges both the design of urban developments to our towns, cities, and villages—and including a life course perspective [48], the SAfE framework is not solely connected to older adults, but younger people too. Finally, the inner sphere—“The age-friendly living environment”—relates to the physical environments of one’s house or apartment, either living on their own or with their families. As noted by Marston and van Hoof [48], this concept has not been previously captured yet. Familial connections and intergenerational relationships are integral to lifestyle, (mental) health and wellbeing, reducing loneliness and social isolation, and enhancing social networks and connectedness.

In the following section, we continue our commentary surrounding age-friendly Blue Zones^®^ frameworks, pulling together a series of recommendations based on the frameworks presented here and offering theoretical insights in an attempt to move the debate and narratives forward.

## 5. Discussion

In this commentary piece, we have presented contemporary literature surrounding age-friendly cities located in the USA, based on the Blue Zones^®^ checklists. We have provided a critical review of the four Blue Zones^®^ checklists and finally we have presented two age-friendly frameworks: 1. the WHO age-friendly framework [3] and 2. the SAfE framework [48], which present different approaches to contemporary society. The second approach instils a nod to the technological revolution which began at the turn of the 21st century and illustrates how technology can and is being used within the age-friendly domain.

Presently, Blue Zones^®^ have proposed four domains (Home, Bedroom, Kitchen and Tribe) with associated checklists [56,57,58,59] comprised of various items and with various motivations. To date, the contemporary literature surrounding Blue Zones^®^ has primarily been from the perspective of health and wellbeing. Yet, there is a paucity of literature surrounding the implementation, use, barriers, enablers, challenges, environmental issues, interventions, and impact surrounding digital technologies in the context of the Blue Zones^®^ checklists and respective regions. Although in the checklists it stipulates that digital technology should kept to a minimum, the 21st century has witnessed and welcomed advances in this area and has not only changed the societal landscape of how we view the use of technology, but also how technology can benefit an individual in all the contexts within these checklists. We have provided examples and suggestions of how technology can be implemented in the home in both the “Kitchen” and “Bedroom” checklists, which may enhance and improve the respective environments should individuals have the financial means to purchase IoT devices and appliances. However, with the implementation of such technological solutions and adaptations, having the digital skillset should also be considered. For many users, old and young, understanding the benefits of purchasing and implementing IoT devices to enhance their living space may not be so evident. While installing the Internet may also be a contentious debate especially if a person is on a low income, this too continues the debate of the digital divide [75]. However, technology and IoT devices cannot be ignored because this area of society has grown phenomenally since the turn of the 21st century and continues to develop and become smarter [48,65,66,67,68].

Worldwide, we observe the growing pervasiveness of digital technologies and services in people’s everyday living and ecosystems. Yet, while the presence of technology is widely acknowledged [48,68], both the Blue Zones^®^ checklists [56,57,58,59] and the WHO age-friendly cities framework [3] devote little attention to this important dimension of societies’ current way of living, respective ecosystems, or consideration for future societies. For example, neither the WHO age-friendly cities framework [3] nor the Blue Zones^®^ checklists [56,57,58,59] have undergone recent iterations and revisions to reflect the changing world. However, the SAfE framework [48] does reflect contemporary society and provides several recommendations to the academy in a bid to move the narrative forward in this growing domain. Similarly, the work presented by Riddle [50] has attempted to illustrate the transferability of the Blue Zones^®^ concepts into Western society across different locations in the USA. Admittingly, for the specific case of the early studies of Blue Zones^®^, this was the case because researchers were focusing on the healthy longevity of people rather than technology, which could be less prominent at the time the research took place. However, this appears to be a weakness, if not a gap, in the later developed checklists and area(s) which now deserves greater investigation and understanding. Serving as a tool to “reengineer” particular locations in the USA after the year 2000, to not account for the presence of digital technologies and services is striking, since neglecting such an important aspect may render the application insufficient.

The Power of 9 [53,54] includes nine items organized into four groups: move, right outlook, eat well, and connect. Although the Blue Zones^®^ checklists also propose an equal number of checklists, there seems to be no correspondence between the two. Critical analysis of the checklists, as presented in Section 4, illustrates the primary goal is outcome, followed by environment and Item 1 in the “Bedroom” checklist transfers to “Intervention delivery”. There are a number of intended goals of the checklists, including: 1. the promotion of physical activity, 2. the motivation towards eating well, and 3. the encouragement of health promotion. However, these three goals fall short of covering all the dimensions of the Power of 9, specifically the nuances of purpose, downshift, and loved ones first. The Home checklist [56] is almost entirely dedicated to encouraging physical activity, with the exception of Item 8 (see Table 1 and Table 7) which seeks to promote air quality, which can also be linked to general health. The Kitchen checklist, [57] is mostly aligned with the eat well dimension, including items that would fall within the three items included in that dimension (the 80% rule, plant slant, and wine at five). Items 4 and 7 of the checklists (see Table 3 and Table 9) also extend the move dimension and general health promotion. The Bedroom checklist [58] (see Table 5 and Table 10) finds no direct correspondence to the Power of 9. It aims at promoting good quality sleep and, in this way, it aims to promote positive health and wellbeing. The Tribe checklist [59] (see Table 7 and Table 11), while designated this way, seems to report on a diverse range of subjects and appears to be an amalgamation of items. This checklist links to general health promotion (Items 4–6, 9) without any direct connection to the Power of 9, physical activity (Item 1), linking to the move dimension of the Power of 9, and an assortment of Items (2, 3, 7–10) could be linked to the right outlook and connect dimensions of the Power of 9. Overall, it is important to underline the underrepresentation of the dimensions: right outlook and connect. As previously noted in earlier sections, population characteristics have not been considered and without this consideration we believe it is difficult to ascertain and fully evaluate the appropriateness of these respective checklists.

Another aspect worth noting is how the Home, Kitchen, and Bedroom checklists [56,57,58] lack theoretical underpinning and as previously noted, we believe taking a life course perspective would benefit future iterations of these checklists greatly because citizens lives and situations change over a course of years and decades, which in turn may impact one’s health and wellbeing. The “Tribe” checklist is subjective and, arguably, difficult to answer and is highly dependent on one’s state of mind or quality over the last few days of an individual. This uneven distribution may lead to a skewed application of the checklist, resulting in an added difficulty to replicate the benefits of the Blue Zones^®^.

The Blue Zones^®^ checklists [56,57,58] somehow touch upon some of the aspects of the WHO age-friendly cities framework [3]—for example, the inclusion of outdoor spaces and buildings (Home checklist), transport (Home checklist), respect and social inclusion (Tribe checklist), social participation (Tribe checklist), community support, and health services (Bedroom checklist). Aspects such as housing, civic participation and employment, communication and information are neglected and still limited. Furthermore, the critical analysis of the checklists highlights that these elements seem to reflect the reality of populations residing in a wealthy developed Western society, characterised by, for example, houses, gardens, and junk food. This way of living may be found in some parts or regions of wealthy developed countries. However, this is not the case everywhere—for example, parts of the population reside in apartments, or even live-in rooms only or multioccupancy housing environments, with no access to gardens or supplementary spaces to implement the checklist items.

All in all, it appears that in aiming to bring Western societies closer to the Blue Zones^®^ principles, with the notion of offering and pursuing a healthier longevity, the checklists [56,57,58,59] have been tailored to a limited segment of society, including individuals and communities who own their own homes, and have few financial worries. This makes the checklists hard to apply in less developed regions or deprived areas of a country, state or county. Future iterations of frameworks should include representative populations from both deprived and affluent areas to gain a complete understanding of how the Power of 9 can increase healthy ageing and longevity.

The various Blue Zones^®^ checklists [56,57,58,59] suggest various amendments to the respective three domains (Home, Kitchen and Bedroom) that could be coupled with lifestyle changes and activities in the fourth domain—Tribe. These checklists do not actually take into consideration the domains outlined in the WHO framework (Figure 1); therefore, building on the WHO framework [3] (Figure 1) and the SAfE framework by Marston and van Hoof [48] (Figure 2) would complement both the checklists and the respective age-friendly frameworks [3,48], better representing the intersect of its multiple components within contemporary 21st century society.

The four Blue Zones^®^ checklists [56,57,58,59] highlight a dearth of items connecting or even considering the application of technology. From a total of 44 items, only 7 items can be connected to technology and the use of power tools and home appliances. Examples in the checklists include, Home, Items 2, 3, and 11 (see Table 1); Kitchen, Item 7 (see Table 3), and Bedroom, Items 5, 7, and 8 (see Table 5). This lack of acknowledgment of technology may pave the way for the limited use and application of the checklists. With this in mind, the SAfE age-friendly framework [48] could provide actors with a basis to bridge future iterations of the Blue Zones^®^ checklists [56,57,58,59] together. Given the SAfE framework [48] illustrates the relationships between the physical space, digital technologies surrounding the living environment and the connections with the respective domains based on the WHO age-friendly framework [3] has the potential to provide a blueprint for this narrative to evolve.

Both the Blue Zones^®^ checklists and the WHO age-friendly cities framework [3] overlook the presence and contribution of technology and associated ICTs. It is hard to imagine a world without technology, even in the Blue Zones^®^ regions where a quick Google search confirms the presence of Internet services and other ICT-related products and services. The important contribution of the environment, physical space, and of technology is stressed in the SAfE framework [48] and offers a good starting point for reflecting on how best to develop an age-friendly physical space and environment in which technology and its associated ICTs can be weaved together, whether it is through assistive devices, smart automation, smart devices, or apps, which act as the connectors between people, physical spaces, and environments in the various Blue Zones^®^.

Besides offering greater coverage of all the presented items as well as the remaining aspects of the Power of 9, future work should explore how the checklists can potentially evolve, taking guidance from the WHO age-friendly cities framework [3] and the SAfE framework [48]. For example, incorporating the Power of 9 into the framework suggested by Marston and van Hoof [48] may be a way to address the inadequacies of the checklists as they are currently presented and to integrate diversity more fully from a variety of perspectives. This future work should be theoretically underpinned by life course theory [76] and implementing an action research approach [77] to ensure all voices and narratives are considered.

### Strengths, Limitations, and Recommendations

This commentary has highlighted an area of social gerontology that has received little attention from scholars who focus on age-friendly and successful age-in-place research. However, given that multi- and cross-disciplinary research is growing, this domain of gerontology—Blue Zones^®^ affords a new area of research for scholars to collaborate and move the narrative forward. This commentary is novel because it highlights gaps in the existing literature and area surrounding the age-friendly domain, Blue Zones^®^ and in this way this innovative piece is a route plan for multi- and cross-disciplinary scholars.

Limitations of this work include the limited evaluation of the checklists [56,57,58,59] and frameworks [3,48]. However, in a recent published paper by Dikken and colleagues [78] who present the Age-Friendly Cities and Communities (AFCC) Questionnaire, there are grounds for existing and future evaluations to take place. This in turn forms the basis and groundwork for future iterations of the AFCC.

As a starting point, we propose that the AFCC survey could be complemented by qualitative data collections such as diaries, fieldtrips, first-hand accounts, interviews, and observations, taking an action research approach [77] to ensure a positive impact upon the respective regions as noted by Marston and colleagues [68]. However, it is possible that the AFCC survey [78] may have to be adapted to accommodate the differing facets of the Blue Zones^®^ regions. This would afford various actors interested in age-friendly and Blue Zones^®^ regions the opportunity to specifically create a supplementary iteration of the survey tailored for this domain(s).

Moreover, an alternative approach to measuring Blue Zones^®^ could consider using quantitative measures by building on the work of Davern and colleagues [79], whereby employing a Geographic Information Systems (GIS) to measure spatial indicators associated with Blue Zones^®^ may afford various actors the opportunity to understand the greater importance and associations reflected in the lived environment(s) in an attempt to facilitate and enhance health and wellbeing. In addition to the work published by Davern et al. [79], Jackisch et al. [80] and the United Nations—New Urban Agenda, the 2030 Agenda for Sustainable Development [81] have also employed GIS techniques and approaches as a means of understanding the impact and importance of spatial indicators associated with age-friendly cities and communities.

Previously, we recommended the implementation of a life course perspective [76], an approach that facilitates researchers, policymakers, educators, students, and communities to view solutions through the lens of personal experiences, and historical events that narrate the story of a “personal biography” [76]. In conjunction with participatory action research [77] approaches, and by implementing universal design principles [82], these methodological approaches will afford scholars to capture and complement future qualitative data collection and narratives from all citizens, not just older people.

Indeed, given the existing five various Blue Zones^®^ regions located worldwide, future frameworks and research should account for the differing cultural aspects, and should be represented in future age-friendly frameworks. Instilling, acknowledging, and embedding cultural beliefs, and traditions is integral to prospective positive implementation and adoption of future age-friendly Blue Zones^®^ frameworks within the respective regions. We believe all four checklists [56,57,58,59] require substantial revisions supported by published evidence-based research from various fields including gerontechnology, geography, ecology, computer science, and social sciences. Given how the “Tribe” checklist [59] appears to be the less developed checklist, we believe this checklist would benefit from a substantial revision. Such a revision should include contemporary measures associated with loneliness [83], technology use [84,85], and environmental factors (e.g., risk of falling) [85,86,87].

Extensive fieldwork encompassing a mixed methods approach is needed to achieve these great strides in this domain and as highlighted by Liddle et al. [77], who purports that localised community engagement is needed to fully understand the specific needs, challenges, barriers, and enablers to social connections, using a bottom-up approach.

Reuter and colleagues [88] have taken a participatory action research approach in their respective work in a bid to understand how older adults and stakeholders use technology to provide digital information and communications. Primarily, Reuter and colleagues [88] focus on two domains featured in the WHO age-friendly framework: 1. communication and information, and 2. civic participation. Furthermore, Marston and colleagues [68] propose implementing universal design principles [82] which, if combined with participatory action research [77] while instilling a bottom-up approach, has the potential to understand the needs, perceptions, expectations, requirements, and impacts of incorporating facets from existing age-friendly frameworks [3,48].

## 6. Conclusions

The aim of this commentary is to outline the initial footprint in a series of future work to bring areas which are limited or lacking in the existing Blue Zones^®^ checklists to the forefront, while also proposing areas for future research within the communities and societies. This is particularly important when we are referring to technology, and globally, we are heading into the third decade of the 21st century.

The authors of this commentary believe this critique is a contribution to the fields of gerontology, gerontechnology, anthropology, geography, and social sciences because, to date, existing research surrounding Blue Zones^®^ regions has primarily focused on the epidemiology and health of citizens, neglecting the opportunity of exploring the social sciences and technological impacts within these regions and looking for an even broader implementation.

We want to open up and welcome further discussions with interested parties, actors, and stakeholders who are interested in age-friendly research, Blue Zones^®^ regions, technology, social sciences, and anthropology, as a way of moving forward with future work, frameworks, and conducting future investigations to advance the knowledge of Blue Zones^®^ regions.

## Figures and Tables

**Table 1:** Questions from the “Home” checklist [56].

Checklist Item	Question	Answer(s)	Points	Purpose/Additional Information
1	Place a scale in a prominent spot in your home and weigh yourself daily. *	I do this now Or I don’t do this	3 points 0 points	“Why do it: People who weigh themselves every day for two years weigh as much as 17 pounds less after two years than people who never weighed themselves. Daily weight checks take only seconds, and the results can provide powerful reinforcement.”
2	Have only one TV in your home. *	I do this now Or I don’t do this	5 points 0 points	How to do it: Have only one TV. Put it in a common room, preferably in a cabinet behind doors. The goal here is to nudge you away from screen time that detracts from physical activity and encourages overeating. Why do it: People who watch too much TV are more likely to be overweight. TV-watching actually lowers metabolism, makes us less active, and encourages us to eat junk food via commercials. Kids with a TV in their bedroom are 18 percent more likely to be (or become) obese and have lower grades. The happiest people watch only 30–60 min of TV per day.
3	Replace power tools with hand tools. *	I do this now Or I don’t do this	5 points 0 points	How to do it: Mow your lawn with a push lawn mower, shovel the snow with a hand shovel, and gather the leaves from your lawn with an old-fashioned rake instead of a leaf blower. Why do it: Shoveling, raking, and push-mowing are healthy and productive outdoor workouts. Some burn almost 400 calories an hour. In fact, mowing the lawn or raking leaves burns about the same number of calories as lifting weights.
4	Grown and maintain your own garden. *	I do this now Or I don’t do this	3 points 0 points	How to do it: Plant a garden in your yard or take a look through the “how-to projects” from the National Gardening Association website (www.garden.org) and choose a project that’s right for you and your space. Start planting and enjoying your delicious produce! Why do it: Gardening is common in all Blue Zones. This activity provides low-intensity range-of-motion exercise, stress reduction, and fresh vegetables. In fact, the CDC points out that you can burn 150 calories by gardening (standing) for approximately 30–45 min.
5	Own a dog. *	I do this now Or I don’t do this	3 points 0 points	How to do it: Take a dog home from your local animal shelter or pet store. However, before you do so, visit the American Kennel Club website to determine if you are ready to commit to a dog and learn how to be a responsible dog owner: http://www.akc.org/public_education/responsible_dog_owneo.cfm. Why do it: Pets make for great companions and encourage you to walk or run. Researchers found that if you own a pet, you get over five hours of exercise a week without a lot of added effort. In fact, studies have shown that dog owners have lower rates of health problems compared to those who don’t own a dog.
6	Own a bicycle (or clean or repair my current bicycle) and a bicycle helmet. *	I do this now Or I don’t do this	5 points 3 points	How to do it: Buy a bike or fix your current bike; then do the same for other family members. Use good quality helmets to prevent injury. Why do it: People who live in Blue Zones areas use active transportation. Not only can you easily incorporate physical activity into your daily life if you own and use a bike, riding at a moderate speed burns approximately 235 calories per half hour. Additionally, wearing a bicycle helmet reduces the risk of serious head injury in crashes by as much as 85% and the risk for brain injury by as much as 88%.
7	Own at least four of the following: basketball, baseball, football, golf balls and clubs, inline skates, camping supplies, running shoes. *	I do this now Or I don’t do this	3 points 0 points	How to do it: Keep sporting equipment nearby to encourage physical activity. Why do it: Owning this equipment makes it easier to practice sports at home. Did you know that inline skating burns more calories than running track and field hurdles and that playing catch for only 30 min burns over 100 calories?
8	Have indoor plants throughout your home. *	I do this now Or I don’t do this	3 points 0 points	How to do it: Pick up some pots, potting soil and some of your favorite greenery to place throughout your home. Golden Pothos Vines and Spider Plants are great starter plants and easy to maintain. Why do it: Did you know that watering plants burns the same amount of calories as stretching or walking? Besides their ability to clean the air, indoor plants have been proven to provide health benefits to people who interact with them. If you keep houseplants, then you’ll be nudged to nurture them daily.
9	Create a destination room. *	I do this now Or I don’t do this	5 points 0 points	How do I do it: Create a room on the top of your home in which you are fully immersed in what you’re doing—where it’s easy to engage in a hobby, read a book, or do a family activity. Include a large table for family projects, shelves filled with books, and plenty of light. Leave out the clock, TV, computer, or other distracting gadgets. Why do it: A popular room on another level of your home increases stair climbing. Did you know that you burn 10 calories per minute climbing up stairs and four calories per minute climbing down them?
10	Disconnect your garage door opener. *	I do this now Or I don’t do this	5 points 0 points	How to do it: Stop using your electric garage door opener. Instead, open the door manually. Why do it: Getting out of the car, raising the door, and returning to the car rather than using a remote control will burn seven calories per minute. Doing this twice a day doesn’t take much time, but will burn extra calories!
11	Create an indoor exercise area. *	I do this now Or I don’t do this	5 points 0 points	How to do it: Designate a portion of a room in your home for your exercise equipment, stability ball, yoga mat, and/or weight set. Why do it: Exercising is made more convenient when you have a space in your home designated for that specific activity. You are more likely to use the equipment it if is easily accessible and visible. A study at the University of Florida found that women who exercised at home lost 25 pounds in 15 months and maintained that loss.
12	Get rid of your TV remote. *	I do this now Or I don’t do this	5 points 0 points	How to do it: Instead of using your TV remote to change the channel, walk over to your TV and manually switch stations. Why do it: Getting up and changing the channel manually 10 times per day will burn 100 calories.
13	Place cushions on the floor. *	I do this now Or I don’t do this	5 points 0 points	How to do it: Instead of sitting on chairs and furniture all the time, sit on cushions on the floor. Why do it: Sitting on the floor works your thighs, glutes, and lower back each time you sit down and stand back up. Supporting yourself without a chair back improves posture and may help you burn up to an additional 130 calories each hour!

* Subscribers to the checklist can enter their email address and receive a copy of their Blue Zones^®^ Home Checklist results.

**Table 2:** Points related to the “Home” checklist [56].

Number of Points	Explanation
55+	Blue Zones Home. You have deconvenienced your living environment in a way that allows you to mindlessly move your way to better health.
30–39	Almost There. You are well on your way to creating an ideal home environment.
15–29	On Your Way. When you begin to pair many of these behaviors together, you’ll start engaging in physical activity more often. Which item is first on your list of changes? Get started on that right now.
Below 15	Just Getting Started. Everyone has to start somewhere. Begin the process by prioritizing the changes you want to make and start on them tomorrow.

**Table 3:** Questions from the “Kitchen” checklist [57].

Checklist Item	Question	Answer(s)	Points	Purpose/Additional Information
1	Package your snacks in proportioned, small bags. *	I do this now Or I don’t do this	5 points 0 points	How to do it: When you buy snacks like pretzels, portion them into small bags to avoid overeating. Why do it: Re-bagging your snacks will help you eat reasonably sized portions. Additionally, you actually burn more calories by preparing fresh meals and snacks.
2	Dedicate the top shelf of your refrigerator to fruits and vegetables. *	I do this now Or I don’t do this	3 points 0 points	How to do it: Get in the habit of keeping your healthy foods on the front of the top shelf of your refrigerator. Why do it: Placing the healthy options at eye level will encourage you to snack mindfully.
3	Only own dinner plates that are 10” or smaller. *	I do this now Or I don’t do this	5 points 0 points	How to do it: Replace your oversized plates with smaller 10” plates. Why do it: Eating smaller plates can promote smaller portions. Over the last 20 years, the average U.S. dinner plate has grown to over 12 inches. During the same timeframe we are eating 22 percent more calories. The easiest, mindless way to eat less is to eat off smaller plates.
4	Drink beverages (except for water) out of smaller glasses. *	I do this now Or I don’t do this	3 points 0 points	How to do it: Replace your big slurp drinking glasses with smaller glasses. Why do it: Larger glasses may increase consumption.
5	Create a junk food drawer. *	I do this now Or I don’t do this	5 points 0 points	How to do it: Put unhealthy snacks and food out of eyes’ reach on bottom shelves or behind cabinet doors. Label it “Junk Food.” Why do it: Most junk food is consumed because you see it and it looks good. If you’re going to have junk food in your house, hiding it from your line of vision will dramatically decrease consumption.
6	Pre-plate your food. *	I do this now Or I don’t do this	5 points 3 points	How to do it: Plate your entire meal before sitting down at the table. Avoid eating family style by leaving the serving dishes on the counter. Why do it: Leave the serving dishes on the counter—not on the table—that way, if you really are hungry for seconds, you’ll be forced to stand up and walk to the kitchen.
7	Remove the TV from your kitchen and dining room. *	I do this now Or I don’t do this	5 points 0 points	How to do it: Remove the TV from your eating environment. Why do it: When other things are going on in your eating environment, you are more likely to pay attention to them rather than the food you are consuming. Avoid multi-tasking while you eat by turning off the TV and radio. Practice this habit while you’re at work, too—try not to work while eating. Take some time away from your desk to eat lunch
8	Put a filled fruit bowl on your countertop. *	I do this now Or I don’t do this	3 points 0 points	How to do it: Take a fruit bowl you already have and put it on your countertop in a well-lit, prominent place. Why do it: Placing the healthy options in convenient, eye-level locations will encourage you to snack mindfully. Keeping the fruit bowl filled will also encourage you to buy a variety of fresh produce items.
9	Use hand operated kitchen appliances. *	I do this now Or I don’t do this	3 points 0 points	How do I do it: Get rid of your electric can opener and use a hand operated one instead. Also get a potato masher and garlic press, rather than an electric mixer. Why do it: Manual kitchen tasks encourage hand and arm strengthening. Try squeezing fruit juice, mashing potatoes or beans, and opening cans manually.
10	Place a longevity food list on your refrigerator. *	I do this now Or I don’t do this	5 points 0 points	How to do it: Create a list with the best longevity foods (nuts, whole grain bread, beans, fruit & vegetables) and the worst junk food (salty snacks, sweetened sugary drinks, processed meats, packaged sweets) and display it on your refrigerator. Why do it: These documents list the best longevity foods to have in your kitchen at all times and the worst junk foods to keep out of your kitchen. They will serve as environmental nudges to help you become more conscious of your consumption.

* Subscribers to the checklist can enter their email address and receive a copy of their Blue Zones^®^ Kitchen Checklist results.

**Table 4:** Points related to the “Kitchen” checklist [57].

Number of Points	Explanation
35+	Blue Zones Kitchen. You have set up your eating environment in a way that allows you to eat healthy meals and snacks. Can you get yourself all the way to scoring 40/40 points?
25–34	Mindful Eater. You are well on your way to creating an ideal eating environment. What other changes are you going to make to have a Blue Zones Kitchen?
15–24	On Your Way. When you begin to pair many of these behaviors together, you’ll start seeing a healthier environment. Which item is first on your list of changes? Get started on that right now.
Below 15	Just Getting Started. Everyone has to start somewhere. Begin the process by prioritizing the changes you want to make and start on them tomorrow.

**Table 5:** Questions from the “Bedroom” checklist [58].

Checklist Item	Question	Answer(s)	Points	Purpose/Additional Information
1	Know your snore score *.	I do this now Or I want to do this	5 points 0 points	How to do it: Determine your snore score by taking the short assessment below. If you answer “yes” to any of the questions, discuss your symptoms with a medical provider. Why do it: The Snore Score was developed by the American Sleep Apnea Association to help individuals assess their risk of sleep apnea, which is a medical condition that can impair sleep and cause health problems. It is important to identify whether sleep problems are due to a medical condition so the condition can be treated early and appropriately. Are you a loud and/or regular snorer? Have you been observed to gasp or stop breathing during sleep? Do you feel tired or groggy upon awakening, or do you awaken with a headache? Are you often tired or fatigued during the wake time hours? Do you fall asleep sitting, reading, watching TV or driving? Do you often have problems with memory or concentration? If you have one or more of these symptoms you are at higher risk for having obstructive sleep apnea. If you are also overweight, have a large neck, and/or have high blood pressure the risk increases even further. If you or someone close to you answers “yes” to any of the above questions, you should discuss your symptoms with your physician or a sleep specialist. Or ask the American Sleep Apnea Association for more information on the diagnosis and treatment of sleep apnea. Different treatment options exist; which is right for you depends upon the severity of your apnea and other aspects of the disorder. Talk to your doctor about choices. Untreated, obstructive sleep apnea can be extremely serious and cannot be ignored. You may also be interested in attending a meeting of an ASAA A.W.A.K.E. group (A.W.A.K.E. stands for “Alert, Well, And Keeping Energetic,” characteristics that are uncommon in people with untreated sleep apnea.) Contact the ASAA for more information about one in your area.
2	Own a comfortable mattress and comfortable pillows *	I do this now Or I want to do this	3 points 0 points	How to do it: Mattresses should be replaced every 8–10 years. Make sure that your mattress is not sagging or not supporting you comfortably during sleep. When choosing a mattress, spend at least 10 min testing it out before buying. Choose pillows that support your head and neck and are comfortable to you. Why do it: Having a comfortable mattress and comfortable pillows are important to getting a good night’s sleep. Getting a good night sleep improves productivity, physical and emotional health, and longevity.
3	Set the temperature in your bedroom to 65 °F at night. *	I do this now Or I want to do this	5 points 0 points	How to do it: Set your thermostat to 65 F at bedtime. If you have a programmable thermostat, program it to automatically adjust to 65 F during sleeping hours. Why do it: Temperatures below 54 F or above 75 F can actually wake you up at night. The ideal temperature for sleep is around 65 F. If it feels a little colder than you’d like, grab a couple of extra blankets.
4	Dim the lights an hour before bed *	I do this now Or I want to do this	3 points 0 points	How to do it: Dim the lights in your home an hour before you go to sleep. Why do it: Practicing good sleep hygiene is the first step to getting the optimal 7–8 h of sleep each night. Dimming the lights before bedtime prepares your body for sleep, allowing you to fall asleep faster and stay asleep longer.
5	Remove digital alarm clocks or turn the clock so it is facing away from the bedside *	I do this now Or I want to do this	3 points 0 points	How to do it: Remove digital alarm clocks from your bedroom or turn your clock away from your bedside so the time is not visible to you. Why do it: The light from digital alarm clocks can disrupt sleep. In addition, hiding your clock from your line of sight will help you avoid clock watching during the night.
6	Hang light blocking window shades in the bedroom *	I do this now Or I want to do this	5 points 3 points	How to do it: Hang dark shades and heavy drapery that can block out all outside light when drawn. Why do it: Light can be disruptive to sleep, even light from a clock or a computer. Make your room as dark as possible for the best sleep.
7	Remove the TV and computer from the bedroom. *	I do this now Or I want to do this	5 points 0 points	How to do it: Remove all screens from your bedroom including televisions, computers and cell phones. Why do it: The bedroom should only be used for sleep and sex. Removing electronic screens from the bedroom helps reinforce the association between the bed and sleep. In addition, artificial light from screens including digital clocks can disrupt sleep.
8	Remove all phones (including cell phones and land line phones) from your bedroom. *	I do this now Or I want to do this	5 points 0 points	How to do it: Remove all phones from the bedroom. Why do it: Removing phones from the bedroom minimizes interruptions to sleep. The 2011 Sleep in America Poll conducted by the National Sleep Foundation found that cell phones were a sleep disturbance. Twenty percent of generation Y’ers and 18% of generation Z’ers polled said that they are awakened after they go to bed by a phone call, text message or email at least a few nights a week.
9	Put a lavender plant next to the bed *	I do this now Or I want to do this	3 points 0 points	How to do it: Purchase a lavender plant from your local florist or sprinkle a little lavender essential oil on your sheets. Why do it: The smell of lavender is calming, soothing, and helps induce sleep.
10	Install double paned windows in the bedroom *	I do this now Or I want to do this	3 points 0 points	How to do it: Install double paned windows in your bedroom. Why do it: Double paned windows help to block out noise, which can be disruptive to sleep. Another way to block out unwanted sounds is to use earplugs or use “white noise” such as a fan, air cleaner or sound conditioner.
11	Use the bedroom only for sleep and sex *	I do this now Or I want to do this	3 points 0 points	How to do it: Avoid doing work, watching TV, using the computer, or doing anything else that might agitate you in your bedroom. Use your bedroom only for sleep and sex. Why do it: Your bedroom environment should be a comfortable and relaxing place that promotes sleep. Avoiding activities that may lead to stress is one way to ensure the bedroom is a place associated with calm and sleep.

* Subscribers to the checklist can enter their email address and receive a copy of their Blue Zones^®^ Bedroom Checklist results.

**Table 6:** Points related to the “Bedroom” checklist [58].

Number of Points	Explanation
35+	Blue Zones Kitchen. You have set up your eating environment in a way that allows you to eat healthy meals and snacks. Can you get yourself all the way to scoring 40/40 points?
25–34	Mindful Eater. You are well on your way to creating an ideal eating environment. What other changes are you going to make to have a Blue Zones Kitchen?
15–24	On Your Way. When you begin to pair many of these behaviors together, you’ll start seeing a healthier environment. Which item is first on your list of changes? Get started on that right now.
Below 15	Just Getting Started. Everyone has to start somewhere. Begin the process by prioritizing the changes you want to make and start on them tomorrow.

**Table 7:** Questions the “Tribe” checklist asks individuals [59].

Checklist Item	Question	Answer(s)	Points
1	In the past month, how many days did you engage in mild or rigorous physical activity (taking stairs, walks, gardening, exercise, etc.)? *	Never Rarely Often	0 points 3 points 5 points
2	During the past month, how often has this person felt sad or depressed? *	Never Rarely Often	5 points 3 points 0 points
3	During the past month, how many days has this person felt lonely? *	Never Rarely Often	5 points 3 points 0 points
4	Does this person smoke? *	No Yes	5 points 0 points
5	Does this person use illegal drugs? *	No Yes	5 points 0 points
6	On average, how many alcoholic drinks does the person have in a typical day? *	None One Two or more	0 points 1 = 3 points 2 or more = 5 points
7	How often does the person participate in social activities? *	Never Once a week More than once a week	0 points 3 points 5 points
8	How often does the person attend religious activities? *	Less than once a week Weekly or more	0 points 5 points
9	Is the person: *	Healthy weight Overweight or obese	5 points 0 points
10	Rate your happiness *	on a scale of 1–10 where 10 represents the best possible life for you and 0 represents the worst possible life for you. What number do you give yourself (or your friends) now?	User inputs their rating into an input box on the website

* Subscribers to the checklist can enter their email address and receive a copy of their Blue Zones^®^ Tribe Checklist results.

**Table 8:** Critical review of the “Home” checklist based on the systematic mapping by Munthe-Kaas et al. [60].

Blue Zone Checklist	Checklist Item	Mapping of Themes								
		Population	Intervention Characteristics	Intervention Delivery	Individual Service Providers	Implementing Organizations	Comparison Intervention	Outcomes	Environmental Context	Researcher Conduct
Home	Place a scale in a prominent spot in your home and weigh yourself daily	-	-	-		-	-	✓	-	-
	Have only one TV in your home	-	-	-	-	-	-	✓	✓	-
	Replace power tools with hand tools	-	-	-	-	-	-	✓	-	-
	Grown and maintain your own garden	-	-	-	-	-	-	✓	-	-
	Own a dog	-	-	-	-	-	-	✓	-	-
	Own a bicycle (or clean or repair my current bicycle) and a bicycle helmet	-	-	-	-	-	-	✓	-	-
	Own at least four of the following: basketball, baseball, football, golf balls and clubs, inline skates, camping supplies, running shoes.	-	-	-	-	-	-	✓	-	-
	Have indoor plants throughout your home	-	-	-	-	-	-	-	✓	-
	Create a destination room	-	-	-	-	-	-	-	✓	-
	Disconnect your garage door opener	-	-	-	-	-	-	✓	✓	-
	Create an indoor exercise area	-	-	-	-	-	-	✓	✓	-
	Get rid of your TV remote	-						✓	-	-
	Place cushions on the floor	-	-	-	-	-	-	✓	✓	-

**Table 9:** Critical review of the “Kitchen” checklist based on the systematic mapping by Munthe-Kaas et al. [60].

Blue Zone Checklist	Checklist Item	Mapping of Themes									
		Population	Intervention Characteristics	Intervention Delivery	Implementation Context	Individual Service Providers	Implementing Organizations	Comparison Intervention	Outcomes	Environmental Context	Researcher Conduct
Kitchen	Package your snacks in proportioned, small bags	-	-	-	-	-	-	-	✓	-	-
	Dedicate the top shelf of your refrigerator to fruits and vegetables	-	-	-	-	-	-	-	✓	✓	-
	Only own dinner plates that are 10” or smaller	-	-	-	-	-	-	-	✓	-	-
	Drink beverages (except for water) out of smaller glasses	-	-	-	-	-	-	-	✓	-	-
	Create a junk food drawer	-	-	-	-	-	-	-	✓	-	-
	Pre-plate your food	-	-	-	-	-	-	-		-	-
	Remove the TV from your kitchen and dining room	-	-	-	-	-	-	-	✓	✓	-
	Put a filled fruit bowl on your countertop	-	-	-	-	-	-	-	✓	-	-
	Use hand operated kitchen appliances	-	-	-	-	-	-	-	✓	-	-
	Place a longevity food list on your refrigerator	-	-	-	-	-	-	-	✓	-	-

**Table 10:** Critical review of the “Bedroom” checklist based on the systematic mapping by Munthe-Kaas et al. [60].

Blue Zone Checklist	Checklist Item	Mapping of Themes								
		Population	Intervention Characteristics	Intervention Delivery	Individual Service Providers	Implementing Organizations	Comparison Intervention	Outcomes	Environmental Context	Researcher Conduct
Bedroom	Know your snore score	-	✓	-	-	-	-	✓	-	-
	Own a comfortable mattress and comfortable pillows	-	-	-	-	-	-	✓	-	-
	Set the temperature in your bedroom to 65 °F at night	-	-	-	-	-	-	-	✓	-
	Dim the lights an hour before bed	-	-	-	-	-	-	-	✓	-
	Remove digital alarm clocks or turn the clock so it is facing away from the bedside	-	-	-	-	-	-	-	✓	-
	Hang light blocking window shades in the bedroom	-	-	-	-	-	-	-	✓	-
	Remove the TV and computer from the bedroom	-	-	-	-	-	-	-	✓	-
	Remove all phones (including cell phones and land line phones) from your bedroom.	-	-	-	-	-	-	-	✓	-
	Put a lavender plant next to the bed	-	-	-	-	-	-	-	✓	-
	Install double paned windows in the bedroom	-	-	-	-	-	-	-	✓	-
	Use the bedroom only for sleep and sex	-	-	-	-	-	-	-	✓	-

**Table 11:** Critical review of the “Tribe” checklist based on the systematic mapping by Munthe-Kaas et al. [60].

Blue Zone Checklist	Check-List Item	Mapping of Themes								
		Population	Intervention Characteristics	Intervention Delivery	Individual Service Providers	Implementing Organizations	Comparison Intervention	Outcomes	Environmental Context	Researcher Conduct
Tribe	In the past month, how many days did you engage in mild or rigorous physical activity (taking stairs, walks, gardening, exercise, etc.)?	-	-	-	-	-	-	✓	-	-
	During the past month, how often has this person felt sad or depressed?	-	-	-	-	-	-	✓	-	-
	During the past month, how many days has this person felt lonely?	-	-	-	-	-	-	✓	✓	-
	Does this person smoke?	-	-	-	-	-	-	✓	-	-
	Does this person use illegal drugs?	-	-	-	-	-	-	✓	-	-
	On average, how many alcoholic drinks does the person have in a typical day?	-	-	-	-	-	-	✓	-	-
	How often does the person participate in social activities?	-	-	-	-	-	-	-	✓	-
	How often does the person attend religious activities?	-	-	-	-	-	-	-	✓	-
	Is the person Healthy weight, overweight or obese	-	-	-	-	-	-	✓	-	-
	Rate your happiness	-	-	-	-	-	-	✓	-	-

## Data Availability

Not applicable.

## References

[1] Van Hoof J., Kazak J.K., Perek-Białas J.M., Peek S.T.M. (2018). The challenges of urban ageing: Making cities age-friendly in Europe. Int. J. Environ. Res. Public Health.

[2] Van Hoof J., Boerenfijn P., Kolmer D.B.G., Marston H.R., Kazak J.K., Verbeek H., Phelan A., O’Shea D. (2020). Chapter 16: Environmental Design for an Ageing Population. Changing Horizons in the 21st Century: Perspectives on Ageing.

[3] World Health Organization (2007). Global Age-Friendly Cities: A Guide.

[4] Plouffe L., Kalache A. (2010). Towards global age-friendly cities: Determining urban features that promote active aging. J. Urban Health.

[5] McDonald B., Walsh K., Scharf T., Buffel T., Handler S., Phillipson C. (2018). Creating an Age-Friendly County in Ireland: Stakeholders’ Perspectives on Implementation. Age-Friendly Cities and Communities: A Global Perspective.

[6] Van Hoof J., Kazak J.K. (2018). Urban ageing. Indoor Built Environ..

[7] Buffel T., Phillipson C. (2016). Can global cities be ‘age-friendly cities’? Urban development and ageing populations. Cities.

[8] Dijkstra L., Poelman H. (2012). Cities in Europe: The New OECD-EC Definition. Regional Focus A Series of Short Papers on Regional Research and Indicators Produced by the Directorate-General for Regional and Urban Policy RF 01/2012. https://ec.europa.eu/regional_policy/sources/docgener/focus/2012_01_city.pdf.

[9] Fitzgerald K.G., Caro F.G. (2014). An Overview of Age-Friendly Cities and Communities Around the World. J. Aging Soc. Policy.

[10] Buffel T., Phillipson C. (2019). Ageing in a Gentrifying Neighbourhood: Experiences of Community Change in Later Life. Sociology.

[11] Peek S.T.M., Wouters E.J.M., van Hoof J., Luijkx K.G., Boeije H.R., Vrijhoef H.J.M. (2014). Factors influencing acceptance of technology for aging in place: A systematic review. Int. J. Med. Inform..

[12] Charness N., Czaja S., Fisk A.D., Rogers W. (2002). Preview Gerontechnology 2002: Creative use of technology for better aging. Gerontechnology.

[13] Tully C.J. (2003). Growing up in technologicalworlds: How modern technologies shape the everyday lives of young people. Bull. Sci. Technol. Soc..

[14] Adams R., Stevenson M., Lang F., Fingerman K. (2004). A Lifetime of Relationships Mediated by Technology. Growing Together: Personal Relationships Across the Life Span.

[15] Barnett K., Adkins B. (2001). Computers: Community for aging women in Australia. Women Environ..

[16] Becker S.A. (2004). A study of Web usability for older adults seeking online health resources. ACM Trans. Comput. Hum. Interact..

[17] Blit-Cohen E., Litwin H. (2004). Elder participation in cyberspace: A qualitative analysis of Israeli retirees. J. Aging Stud..

[18] Bradley N., Poppen W. (2003). Assistive technology, computers and Internet may decrease sense of isolation for homebound elderly and disabled persons. Technol. Disabil..

[19] Cody M.J., Dunn D., Hoppin S., Wendt P. (1999). Silver surfers: Training and evaluating Internet use among older adult learners. Commun. Educ..

[20] Dickinson A., Gregor P. (2006). Computer use has no demonstrated impact on the well-being of older adults. Int. J. Hum. Comput. Stud..

[21] Hargittai E. (2007). Whose space? Differences among users and non-users of social network sites. J. Comput. Mediat. Commun..

[22] Hargittai E., Shafer S. (2006). Differences in actual and perceived online skills: The role of gender. Soc. Sci. Q..

[23] Kanayama T. (2003). Ethnographic research on the experience of Japanese elderly people online. New Media Soc..

[24] Kavanaugh A.L., Patterson S.J. (2001). The impact of community computer networks on social capital and community involvement. Am. Behav. Sci..

[25] Laguna K., Babcock R.L. (1997). Computer anxiety in young and older adults: Implications for human–computer interactions in older populations. Comput. Hum. Behav..

[26] McConatha D., McConatha J.T., Dermigny R. (1994). The use of interactive computer services to enhance the quality of life for long-term care residents. The Gerontologist.

[27] McMellon C.A., Schiffman L.G. (2002). Cybersenior empowerment: How some older individuals are taking control of their lives. J. Appl. Gerontol..

[28] Melenhorst A.S., Rogers W.A., Bouwhuis D.G. (2006). Older adults’ motivated choice for technological innovation: Evidence for benefit-driven selectivity. Psychol. Aging.

[29] Ajala O., English P., Pinkney J. (2013). Systematic Review and Meta-Analysis of Different Dietary Approaches to the Management of Type 2 Diabetes. Am. J. Clin. Nutr..

[30] Beezhold B.L., Johnston C.S., Daigle D.R. (2010). Vegetarian Diets Are Associated With Healthy Mood States: A Cross-Sectional Study in Seventh-Day Adventist Adults. Nutr. J..

[31] Chrysohoou C., Panagiotakos D.B., Aggelopoulos P., Kastorini C.M., Kehagia I., Pitsavos C., Stefanadis C. (2010). The Mediterranean Diet Contributes to the Preservation of Left Ventricular Systolic Function and to the Long-Term Favorable Prognosis of Patients Who Have Had an Acute Coronary Event. Am. J. Clin. Nutr..

[32] Chrysohoou C., Pitsavos C., Panagiotakos D., Skoumas J., Lazaros G., Oikonomou E., Galiatsatos N., Striggou M., Xynogala M., Stefanadis C. (2013). Long-Term Fish Intake Preserves Kidney Function in Elderly Individuals: The Ikaria Study. J. Ren. Nutr..

[33] Darmadi-Blackberry I., Wahlqvist M.L., Kouris-Blazos A., Steen B., Lukito W., Horie Y., Horie K. (2004). Legumes: The most important dietary predictor of survival in older people of different ethnicities. Asia Pac. J. Clin. Nutr..

[34] Ford P.A., Jaceldo-Siegl K., Lee J.W., Youngberg W., Tonstad S. (2013). Intake of Mediterranean foods associated with positive affect and low negative affect. J. Psychosom. Res..

[35] Fraser G.E. (2009). Vegetarian diets: What do we know of their effects on common chronic diseases?. Am. J. Clin. Nutr..

[36] Antonogeorgos G., Panagiotakos D.B., Pitsavos C., Papageorgiou C., Chrysohoou C., Papadimitriou G.N., Stefanadis C. (2012). Understanding the role of depression and anxiety on cardiovascular disease risk, using structural equation modeling; the mediating effect of the Mediterranean diet and physical activity: The ATTICA study. Ann. Epidemiol..

[37] Bazzano L.A., He J., Ogden L.G., Loria C., Vupputuri S., Myers L., Whelton P.K. (2001). Legume consumption and risk of coronary heart disease in US men and women: NHANES I Epidemiologic Follow-up Study. Arch. Intern. Med..

[38] Bazzano L.A., Thompson A.M., Tees M.T., Nguyen C.H., Winham D.M. (2011). Non-soy legume consumption lowers cholesterol levels: A meta-analysis of randomized controlled trials. Nutr. Metab. Cardiovasc. Dis..

[39] Chrysohoou C., Skoumas J., Pitsavos C., Masoura C., Siasos G., Galiatsatos N., Psaltopoulou T., Mylonakis C., Margazas A., Kyvelou S. (2011). Long-term adherence to the Mediterranean diet reduces the prevalence of hyperuricaemia in elderly individuals, without known cardiovascular disease: The Ikaria study. Maturitas.

[40] Clarkson T.B. (2002). Soy, Soy Phytoestrogens, and Cardiovascular Disease. J. Nutr..

[41] Covas M.I., Konstantinidou V., Fitó M. (2009). Olive oil and cardiovascular health. J. Cardiovasc. Pharmacol..

[42] Fontana L., Villareal D.T., Weiss E.P., Racette S.B., Steger-May K., Klein S., Holloszy J.O., Washington University School of Medicine CALERIE Group (2007). Calorie restriction or exercise: Effects on coronary heart disease risk factors. A randomized, controlled trial. Am. J. Physiol. Endocrinol. Metab..

[43] Carru C., Pes G.M., Deiana L., Baggio G., Franceschi C., Lio D., Balistreri C.R., Candore G., Colonna-Romano G., Caruso C. (2003). Association Between the HFE Mutations and Longevity: A Study in Sardinian Population. Mech. Ageing Dev..

[44] Caselli G., Lipsi R.M. (2006). Survival Differences Among the Oldest Old in Sardinia: Who, What, Where, and Why?. Demogr. Res..

[45] Caselli G., Pozzi L., Vaupel J.W., Deiana L., Pes G., Carru C., Franceschi C., Baggio G. (2006). Family clustering in Sardinian longevity: A genealogical approach. Exp. Gerontol..

[46] Davinelli S., Willcox D.C., Scapagnini G. (2012). Extending healthy ageing: Nutrient sensitive pathway and centenarian population. Immun. Ageing.

[47] Christakis N.A., Fowler J.H. (2007). The spread of obesity in a large social network over 32 years. N. Engl. J. Med..

[48] Marston H.R., van Hoof J. (2019). Who Doesn’t Think about Technology When Designing Urban Environments for Older People? A Case Study Approach to a Proposed Extension of the WHO’s Age-Friendly Cities Model. Int. J. Environ. Res. Public Health.

[49] Buettner D. (2015). The Blue Zones Solution: Eating and Living Like the World’s Healthiest People.

[50] Riddell B. (2016). Blue Zones: Rethinking the American Landscape. School of City and Regional Planning, College of Architecture, Georgia Institute of Technology. https://smartech.gatech.edu/bitstream/handle/1853/55168/briana_riddell_blue_zones_rethinking_the_american_landscape.pdf.

[51] Albert Lea, Minnesota. Wikipedia. https://en.wikipedia.org/wiki/Albert_Lea,_Minnesota.

[52] Spence, Iowa. Wikipedia. https://en.wikipedia.org/wiki/Spencer,_Iowa.

[53] Reverse Engineering Longevity. https://www.bluezones.com/2016/11/power-9/.

[54] Buettner D. (2015). Power 9^®^.

[55] Blue Zones Registration. https://www.bluezones.com/live-longer-better/checklists/registration/?redirect_to=https%3A%2F%2Fwww.bluezones.com%2Flive-longer-better%2Fchecklists%2F.

[56] Home Checklist. https://www.bluezones.com/live-longer-better/checklists/checklist-home/.

[57] Kitchen Checklist. https://www.bluezones.com/live-longer-better/checklists/kitchen/.

[58] Bedroom Checklist. https://www.bluezones.com/live-longer-better/checklists/bedroom/.

[59] Tribe Checklist. https://www.bluezones.com/live-longer-better/checklists/tribe/.

[60] Munthe-Kaas H., Nøkleby H., Nguyen L. (2019). Systematic mapping of checklists for assessing transferability. Syst. Rev..

[61] Hadley R.A. (2020). Men and Me(n). Methodol. Innov..

[62] Hadley R.A., Sparkes A.C. (2018). The Lived Experience of Older Involuntary Childless Men. The Annual Journal of the British Sociological Association Study Group on Auto/Biography.

[63] Hadley R.A., Westwood S. (2018). Ageing Without Children, Gender and Social Justice. Ageing, Diversity and Equality: Social Justice Perspectives.

[64] Hadley R.A., Barry J., Kingerlee R., Seager M., Sullivan L. (2019). Deconstructing Dad. The Palgrave Handbook of Male Psychology and Mental Health.

[65] Smarter. AM. https://smarter.am/.

[66] Pantri. https://pantri.net/.

[67] Marston H.R., Samuels J. (2019). A Review of Age Friendly Virtual Assistive Technologies and their Effect on Daily Living for Carers and Dependent Adults. Healthcare.

[68] Marston H.R., Shore L., White P.J. (2020). How does a (Smart) Age-Friendly Ecosystem Look in a Post-Pandemic Society?. Int. J. Environ. Res. Public Health.

[69] BBC News (2018). Amazon Echo Trial to Help Elderly and Disabled People. https://www.bbc.co.uk/news/av/uk-politics-43869120/amazon-echo-trial-to-help-elderly-and-disabled-people.

[70] Hampshire County Council (2018). Hampshire County Council: Pushing the Boundaries by Using Amazon Echo. https://www.local.gov.uk/hampshire-county-council-pushing-boundaries-using-amazon-echo.

[71] Peskett J. (2019). Virgin Launches New Alexa Initiative to Assist Disabled Passengers. Accessibility Mobility Professional. https://www.accessandmobilityprofessional.com/virgin-launches-new-alexa-initiative-to-assist-disabled-passengers/.

[72] Buffel T., Phililipson C., Rémillard-Boilard S., Gu D., Dupre M.E. (2019). Age-Friendly Cities and Communities: New Directions for Research and Policy. Encyclopedia of Gerontology and Population Aging.

[73] Van Hoof J., Dikken J., Buttiġieġ S.C., van den Hoven R.F.M., Kroon E., Marston H.R. (2020). Age-friendly cities in The Netherlands: An explorative study of facilitators and hindrances in the built environment and ageism in design. Indoor Built Environ..

[74] Rowles G. (1983). Place and identity in old age. J. Exp. Psychol..

[75] Marston H.R. (2019). Millennials and ICT—Findings from the Technology 4 Young Adults (T4YA) Project: An Exploratory Study. Societies.

[76] Elder G.H. (1985). Perspectives on the Life Course. Life Course Dynamics: Trajectories and Transitions, 1968–1980.

[77] Liddle J., Pitcher N., Montague K., Hanratty B., Standing H., Scharf T. (2020). Connecting at Local Level: Exploring Opportunities for Future Design of Technology to Support Social Connections in Age-friendly Communities. Int. J. Environ. Res. Public Health.

[78] Dikken J., van den Hoven R.F., van Staalduinen W.H., Hulsebosch-Janssen L.M., van Hoof J. (2020). How Older People Experience the Age-Friendliness of Their City: Development of the Age-Friendly Cities and Communities Questionnaire. Int. J. Environ. Res. Public Health.

[79] Davern M., Winterton R., Brasher K., Woolcock G. (2020). How Can the Lived Environment Support Healthy Ageing? A Spatial Indicators Framework for the Assessment of Age-Friendly Communities. Int. J. Environ. Res. Public Health.

[80] Jackisch J., Zamaro G., Green G., Huber M. (2015). Is a healthy city also an age-friendly city?. Health Promot. Int..

[81] United Nations (2015). Transforming out World: The 2030 Agenda for Sustainable Development.

[82] NSAI (2019). EN 17161:2019 Design for All. Accessibility Following a Design for all Approach in Products, Goods and Services. Extending the Range of Users. In Dublin, Ireland: National Standards Authority of Ireland. https://www.nsai.ie/about/news/a-design-standard-that-works-for-all/.

[83] De Jong Gierveld J., Van Tilburg T. (2006). A 6-Item Scale for Overall, Emotional, and Social Loneliness: Confirmatory Tests on Survey Data. Res. Aging.

[84] Marston H.R., Genoe R., Freeman S., Kulczycki C., Musselwhite C. (2019). Older Adults’ Perceptions of ICT: Main Findings from the Technology in Later Life (TILL) Study. Healthcare.

[85] Vaziri D.D., Aal K., Ogonowski C., Von Rekowski T., Kroll M., Marston H.R., Poveda R., Gschwind Y.J., Delbaere K., Wieching R. (2016). Exploring user experience and technology acceptance for a fall prevention system: Results from a randomized clinical trial and a living lab. Eur. Rev. Aging Phys. Act..

[86] Marston H.R., Woodbury A., Gschwind Y.J., Kroll M., Fink D., Eichberg S., Kreiner K., Ejupi A., Annegarn J., de Rosario H. (2015). The design of a purpose-built exergame for fall prediction and prevention for older people. Eur. Rev. Aging Phys. Act..

[87] Delbaere K., Smith S.T., Lord S.R. (2011). Development and initial validation of the Iconographical Falls Efficacy Scale. J. Gerontol. Ser. A.

[88] Reuter A., Liddle J., Scharf T. (2020). Digitalising the Age-Friendly City: Insights from Participatory Action Research. Int. J. Environ. Res. Public Health.

